# Supplementation of DHA enhances the cryopreservation of yak semen via alleviating oxidative stress and inhibiting apoptosis

**DOI:** 10.3389/fvets.2025.1532473

**Published:** 2025-02-26

**Authors:** Yanjin Zhu, Jun Yu, Xupeng Li, Zhuo Chen, Yuan Li, Yan Xiong, Honghong He, Shi Yin, Daoliang Lan, Jian Li, Lixue Yang, Xianrong Xiong

**Affiliations:** ^1^Key Laboratory for Animal Science of National Ethnic Affairs Commission, Southwest Minzu University, Chengdu, China; ^2^Reproductive Medicine Center, The Third People's Hospital of Chengdu, Chengdu, China; ^3^Key Laboratory of Qinghai-Tibetan Plateau Animal Genetic Resource Reservation and Exploitation of Ministry of Education, Southwest Minzu University, Chengdu, China

**Keywords:** yak, sperm, cryopreservation, DHA, antioxidant

## Abstract

**Introduction:**

Semen cryopreservation is a crucial method for preserving genetic resources and accelerating the breeding process in domestic animals. However, the frozen-thawed process often leads to physical and chemical damage in semen, resulting in oxidative stress that diminishes sperm vitality and fertilization potential. This study aimed to explore the effects of docosahexaenoic acid (DHA) on the quality of frozen-thawed yak semen.

**Methods:**

Semen samples were collected from six healthy adult Maiwa yaks and cryopreserved in liquid nitrogen using extenders with varying DHA concentrations: 0, 0.1, 1, 10, and 100 ng/mL. After thawing, we assessed indices, antioxidant capacity, mitochondrial activity, and apoptosis status to identify the optimal DHA concentration.

**Results and discussion:**

Our findings indicate that the addition of DHA significantly improved the total motility (TM), progressive motility (PM), velocity of straight line (VSL), curvilinear velocity (VCL), and average path velocity (VAP) of cryopreserved spermatozoa, as well as the integrity of membrane and acrosome (*P* < 0.05). Additionally, DHA supplementation markedly reduced the levels of reactive oxygen species (ROS) and malondialdehyde (MDA) in frozen-thawed yak spermatozoa (*P* < 0.05) and enhanced the antioxidant enzyme activities (T-AOC, SOD, CAT, GSH-Px, *P* < 0.05). It also improved the mitochondrial membrane potential (MMP) and ATP levels (*P* < 0.05). Notably, the group treated with 10 ng/mL DHA showed significantly better outcomes than the other treatment groups (*P* < 0.05). Furthermore, the addition of 10 ng/mL DHA to the semen cryopreservation dilution effectively decreased the apoptotic ratio of frozen-thawed yak spermatozoa (*P* < 0.05), and notably upregulated the expression level of anti-apoptotic protein Bcl-2 (*P* < 0.05), while downregulating the expression of the pro-apoptotic protein Bax and Caspase3 (*P* < 0.05).

**Conclusion:**

In conclusion, the incorporation of 10 ng/mL DHA into semen extenders enhances the quality and viability of yak sperm after cryopreservation by alleviating the oxidative stress, bolstering antioxidant defenses, and preserving mitochondria function, as well as inhibiting the apoptotic pathway activation.

## 1 Introduction

The yak (*Bos grunniens*), a quintessential livestock species, predominantly inhabits the Qinghai-Tibet Plateau at 3,500 m above sea level. These animals exhibit remarkable adaptability to the harsh alpine and hypoxic conditions, serving as a vital resource for local inhabitants by providing meat, milk, fur, and transportation (1). However, the yak's growth and reproductive performance are comparatively lower than those of the other cattle breeds due to the extreme environmental conditions they endure (2). To enhance the efficient utilization of yak, artificial insemination (AI) techniques have been employed, crossbreeding them with cattle possessing superior traits. Semen cryopreservation, a fundamental aspect of AI, is crucial for the conservation of genetic resources and the success of breeding programs.

The overarching principle of semen preservation involves reducing the metabolic activity of sperm to extend their lifespan. For instance, cryopreservation at −196°C in liquid nitrogen halts sperm activity, thereby preserving their viability (3). However, both physical and chemical damage to sperm can occur during cryopreservation due to crystallization, leading to oxidative damage (4). Reactive oxygen species (ROS), such as superoxide anions (O^2−^), are primarily generated by the interaction of electrons with oxygen at complexes I and III of the mitochondrial electron transport chain (ETC) (5). While ROS levels in normal biological processes do not affect sperm, cryopreservation can alter the sperm's physiochemical properties, leading to excessive ROS production. This, in turn, can damage the mitochondrial membrane and trigger continuous ROS generation (6). Oxidative stress can diminish sperm motility, ATP production, and mitochondrial activity, and it can also impair the mitochondrial transcription system (7). This impairment is largely due to nuclear or mitochondrial DNA damage caused by oxidative stress, which hinders the replication and transcription of genes involved in energy metabolism, thereby reducing sperm motility (8). Consequently, maintaining sperm mitochondrial function after freezing and thawing is essential for preserving the fertilization potential of sperm during cryopreservation and for enhancing the efficiency of AI. Previous studies have shown that the addition of antioxidants to semen extenders alleviates oxidative stress during cryopreservation (9–11).

Essential polyunsaturated fatty acids (PUFAs), which are integral to animal nutrition, cannot be synthesized endogenously. Among these, the ω-3 family, including eicosapentaenoic acid (EPA, 20:5 n-3) and docosahexaenoic acid (DHA, 22:6 n-3), have been shown to significantly influence the structure and function of mammalian cells (12). Research has demonstrated the antioxidant, anti-inflammatory, and cardiovascular properties of DHA and other n-3 PUFAs (13–16). Studies on IMS32 cells revealed that DHA and EPA active antioxidant enzymes through Nrf2 transcription to combat oxidative stress (17). Furthermore, research on injured brain tissue has shown that DHA can alleviate mitochondrial dysfunction, reduce ROS and MDA levels, enhance SOD content to improve cell viability, and regulate the apoptosis-related genes to alleviate cell apoptosis caused by oxidative stress (18). As an exogenous cryoprotectant in the semen extender, DHA has been shown to maintain the quality and fertilization ability of frozen trout sperm and to effectively improve the motility of frozen-thawed bull spermatozoa (19, 20). However, the application of DHA in yak semen cryopreservation has rarely been reported.

Currently, yak semen cryopreservation technology and commercial cryoprotectants are relatively backward, resulting in lower-quality frozen semen than that of other species. Studies have indicated that sperm are susceptible to oxidative stress during cryopreservation, leading to apoptosis, and the addition of antioxidants to sperm extenders can effectively alleviate this damage (18–20). As a common antioxidant, the effects of DHA on yak semen cryopreservation have been seldom explored. Thus, this study aims to evaluate the impact of DHA on the kinematic performance, structural integrity, antioxidant capacity, mitochondrial function, and apoptosis status of yak spermatozoa. The goal is to determine the optimal concentration of DHA in yak semen extenders and to explore the underlying mechanisms, providing a theoretical foundation for the development of improved yak semen cryopreservation systems and cryoprotectants.

## 2 Materials and methods

### 2.1 Semen collection and quality assay

All experimental procedures were conducted in strict accordance with the guidelines of the Southwest Minzu University Academic Committee (Approval code, SMU-CAVS-240530008). Semen samples were collected from six healthy, adult Maiwa yaks, aged 4 years old (free grazing on grasslands), using electroejaculation during the breeding season for three times, which spans from July to August. The collection site was located in Hongyuan County, China, at an altitude of ~3,500 m, with 102°34′E longitudinal and 32°47′N latitudinal. Immediately following collection, the fresh semen was transported to the local laboratory in a thermos flask containing water maintained at 37°C. There, the motility and concentration of the semen were visually assessed using a computer-assisted sperm analysis system (CASA, AndroVision, Minitube Co., Germany). Only samples exhibiting total motility exceeding 90% were selected and pooled for further analysis in this study.

### 2.2 Experimental design and semen processing

Qualified fresh yak semen was gently mixed and extended with a pre-heated (37°C) commercial semen diluent (Optidyl, Fleurance, France) at a ratio of 1:4 (v/v). This mixture was then divided into five groups and supplemented with semen diluent containing 0, 0.1, 1, 10, and 100 ng/ml DHA respectively. Subsequently, all semen groups were gradually cooled to 4°C and held at this temperature for 1 h to allow for equilibration. The cooled semen samples were then carefully loaded into 0.25 ml straws (IMV Technologies, France). These straws were subsequently fumigated by exposure to vapors ~10–15 cm above the surface of liquid nitrogen for 15 min. After fumigation, the straws were rapidly immersed in a liquid nitrogen tank for long-term storage.

Following a 1-week storage period, straws from all treatment groups were rapidly thawed in a 42°C water bath for 30 s. The semen was then carefully transferred into brown centrifuge tubes for further analysis and trials. This meticulous process ensured the preservation of semen quality and the integrity of subsequent experimental procedures.

### 2.3 Spermatozoa motility and kinematics analysis

Post-thawing, the sperm motility and kinematics parameters of the five treatment groups were assessed. The procedure involved placing 2 μl of each semen sample on a pre-heated glass slide, covered with a cover slip to create a uniform layer for analysis. The motion tracks of yak sperm were captured using the CASA system, a state-of-the-art tool for sperm motility assessment. The CASA system evaluated several key motility parameters, including total motility (TM), progressive motility (PM), straight-line velocity (VSL), curvilinear velocity (VCL), and average path velocity (VAP). These parameters provide a comprehensive assessment of sperm motility, which is crucial for determining sperm function and potential fertility. For each sample, at least 10 different fields of view were inspected under 200× magnification, ensuring the analysis included more than 200 spermatozoa. This rigorous sampling strategy allowed for a reliable and accurate assessment of sperm motility and kinematics across the different treatment groups. By examining a large number of sperm cells, the study aimed to obtain statistically significant data that accurately reflects the effects of varying DHA concentrations on sperm quality post-cryopreservation.

### 2.4 Acrosome integrity analysis

Acrosome integrity, a critical factor for sperm function, was evaluated using fluorescein isothiocyanate-peanut agglutinin (FITC-PNA, Sigma-Aldrich, MO, USA) staining combined with Hoechst 33342, with minor modification as previous described (21, 22). Hoechst 33342 is a fluorescence dye that binds to the nucleus, emitting blue fluorescence and allowing for the location of sperm cells. In contrast, FITC-PNA specifically binds to the intact acrosome of sperm, emitting a crescent-shaped green fluorescence that overlays the blue fluorescence. The staining procedure began with smearing 10 μl of semen sample onto a microscope slide, allowing it to air-dry, and then fixing it with methanol for 10 min. The slide was then covered with a mixture of 60 μl FITC-PNA and Hoechst 33342 (1:1, v/v) and incubated for 30 min at 37°C in the dark to allow for optimal staining. After incubation, the slides were gently washed with PBS, air-dried, and then observed under a fluorescence microscope at 400× magnification. Sperm with blue nuclei and green-crescent-shaped acrosomes were classified as having intact acrosomes, while those lacking green caps were considered to have damaged acrosomes. The ratio of intact sperm acrosomes to the total number of identifiable spermatozoa (blue dots with and without green caps) was determined by randomly selecting 10 fields of view and using the CASA system to record and calculate the percentage of acrosomes integrity across the different treatment groups.

### 2.5 Membrane integrity assay

Membrane integrity was assessed using a dual-staining method with Hoechst 33342/PI (Meilunbio, Liaoning, China). Intact sperm cells are permeable to Hoechst 33342, which stains the nucleus and emits a blue fluorescence. Conversely, PI penetrates cells with compromised membranes, intercalating with DNA and producing a red or pink fluorescence that overlays the blue fluorescence. Following counterstain, 10 μl of the stained semen sample was smeared onto a slide and examined under a fluorescence microscope (Olympus, Tokyo, Japan) at 400× magnification. Spermatozoa with intact membranes appeared as blue fluorescence dots, while those with damaged membranes appeared as pink or red dots. To quantify membrane integrity, at least 10 random fields of view were selected from each slide, and the percentages of blue and pink/red dots were counted using the CASA system.

### 2.6 Evaluation of ROS level

The levels of ROS were determined using a commercial ROS assay kit (R6033, UElandy, Jiangsu, China), following the manufacturer's protocol. This assay employs the non-fluorescent 2′,7′-Dichlorodihydrofluorescein diacetate (DCFH-DA), which exhibits green fluorescence. After incubating with the dye, semen samples were washed to remove any unbound probes and then prepared as smears for analysis. The slides were examined under a fluorescence microscope (Olympus, Tokyo, Japan) at 630× magnification to visually assess the overall ROS production within the sperm cells. The density of the fluorescence intensity was measured using a multi-mode microplate reader (SpectraMax iD3, Molecular Devices, USA). To normalize the data, the relative fluorescence density of each sample was adjusted based on the average intensity of the control group at 0 h.

### 2.7 MDA content detection

MDA concertation was assessed using a commercial MDA kit (A003-1-2, Jiancheng, Jiangsu, China) according to the manufacturer's instructions. Briefly, ~10^6^ spermatozoa were collected and disrupted with an ultrasonic homogenizer (SCIENTZ-IID, Scientz, Zhejiang, China) to fragment the cell membrane. Following the disruption, the sample was diluted with PBS and mixed with the reaction reagents. Then the mixture was incubated in a boiling water bath for 40 min to allow for the development of the color reaction. Finally, the samples were rapidly cooled in a stream of flowing water to terminate the reaction. The absorbance of the samples was subsequently measured using a spectrophotometer (MultiskanSky, Thermo Scientific, Shanghai, China) at a wavelength of 532 nm. The MDA concentrations were expressed as nmol/ml, offering a standardized representation of lipid peroxidation levels within the sperm samples.

### 2.8 Detection of antioxidant capacity

To evaluate the antioxidant capacity of frozen-thawed yak spermatozoa, several commercial assay kits were employed to measure different components of the antioxidant system. Specifically, T-AOC assay kit (A015-3-1, Jiancheng, Jiangsu, China), SOD assay kit (A001-3-2, Jiancheng, Jiangsu, China), CAT assay kit (A007-1-1, Jiancheng, Jiangsu, China) and GSH-Px assay kit (S0056, Beyotime, Shanghai, China) were utilized. Briefly, spermatozoa were collected by centrifugation, followed by resuspension in PBS. The sperm cells were then disrupted using an ultrasonic homogenizer Ultrasonic Homogenizer (SCIENTZ-IID, Scientz, Zhejiang, China) to ensure the release of intracellular antioxidants. After that, the samples were centrifuged at 12,000 rpm for 5 min at 4°C and the supernatant was collected. The following operation and detection of each index were in accordance with the manufacturer's instructions. The absorbance of each sample was measured using a spectrophotometer (MultiskanSky, Thermo Scientific, Shanghai, China) at a specific wavelength tailored to each antioxidant assay. The concentrations of antioxidants in each sample were calculated based on the standard curves provided with the assay kits.

### 2.9 ATP content assay

To determine the ATP concentrations in the semen samples, an ATP assay kit (A095-1-1, Jiancheng, Jiangsu, China) was used. The spermatozoa were collected by centrifugation, and then the sperm cells were resuspended in 0.9% saline. To ensure complete cell lysis and the release of ATP, the sperm suspension was disrupted using an ultrasonic homogenizer Ultrasonic Homogenizer (SCIENTZ-IID, Scientz, Zhejiang, China). The sperm suspensions were boiled for 10 min to further ensure cell disruption and ATP release. After boiling, the samples were vortexed for 1 min to create a homogeneous mixture, then mixed with the reagent and processed as per the manufacturer's instructions. Finally, the absorbance of all samples was measured using a spectrophotometer (MultiskanSky, Thermo Scientific, Shanghai, China) at a wavelength of 636 nm and the ATP contents were calculated based on the instructions.

### 2.10 Detection of mitochondrial membrane potential

The MMP was assessed using a commercial JC-1 mitochondrial membrane potential detection kit (J6004L, UElandy, Jiangsu, China), following the manufacturer's instructions. Briefly, under conditions of high mitochondrial membrane potential, JC-1 accumulates in the mitochondrial matrix and forms red fluorescent polymers. While under low MMP conditions, JC-1 remains green fluorescent monomers. The fluorescence of the mitochondrial staining was visualized using a fluorescence microscope (Olympus, Tokyo, Japan) at 400× magnification. The intensity of the fluorescence intensity was measured using a multi-mode microplate reader (SpectraMax iD3, Molecular Devices, USA). The MMP was calculated as the ratio of red and green fluorescence intensity, which provides a quantitative measure of the MMP across the different treatment groups.

### 2.11 Annexin V-FITC/PI assay

To evaluate the apoptosis status of frozen-thawed yak spermatozoa, the Annexin V-FITC/PI double staining apoptosis detection kit (G003-1, Jiancheng, Jiangsu, China) was utilized following the manufacturer's instructions. Simply, after removing the extender and resuspending by PBS, the spermatozoa were mixed with a binding solution. Following that the sperm samples were incubated with the Annexin V-FITC/PI working solution for 10 min at room temperature in the dark. After incubation, 5 μl of the sperm mixture was used to make smears on slides. The apoptosis status of the spermatozoa was examined using a fluorescence microscope (Olympus, Tokyo, Japan) at 400× magnification. Sperm cells exhibiting green fluorescence were indicative of early apoptosis, those showing both green and red fluorescence were indicative of late apoptosis, and those stained only red were considered non-viable. Live sperm cells, which maintain membrane integrity and do not expose phosphatidylserine, showed no fluorescence. Ten random fields of spermatozoa were acquired for each sample, and the number of spermatozoa in each apoptotic category was recorded.

### 2.12 Western blot

The sperm protein expressions of apoptosis-related pathways were detected after semen cryopreservation. The thawed yak semen samples were centrifuged to remove the extender and the sperm proteins were extracted using the Total Protein Extraction Kit (BC3710, Solarbio, Beijing, China). After detecting the protein concentrations using the BCA Protein Assay Kit (PC0020, Solarbio, Beijing, China), all the samples were diluted with 5× loading buffer (v:v = 4:1) and boiled for 10 min. The total proteins were separated by sodium dodecyl sulfate-polyacrylamide gel electrophoresis (SDS–PAGE, P0012AC, Beyotime, Shanghai, China). Then the proteins were transferred onto the polyvinylidene fluoride (PVDF) membrane (FFP22, Beyotime, Shanghai, China), subsequently blocked with 5% non-fat powdered milk for 2 h at room temperature. The PVDF membranes with protein blots were incubated with specific primary antibodies against Bax (1:1,000, AF0120, Affinity, Jiangsu, China), Bcl-2 (1:1,000, AF6139, Affinity, Jiangsu, China), Cleaved-Caspase3 antibody (1:1,000, AF7022, Affinity, Jiangsu, China), and β-actin (1:10,000, AF7018, Affinity, Jiangsu, China) overnight at 4 °C. After washing three times with Tris-buffered saline containing 0.1% Tween-20 (TBST), the PVDF membranes were incubated with HRP conjugated goat anti-rabbit IgG (H + L) secondary antibody (1:5,000, S0001, Affinity, Jiangsu, China) for 2 h at room temperature. The PVDF membranes were washed with TBST three times and then visualized using the BeyoECL Star Kit (P0018AS, Beyotime, Shanghai, China). The blotting images were captured using the iBright imaging system (CL1000, Thermo Fisher, USA). The gray values of blotting were analyzed by ImageJ software (National Institutes of Health, USA).

### 2.13 Statistical analysis

The analysis of the raw data was conducted using SPSS 19.0 (SPSS Inc., Chicago, IL, USA) software with two-way analysis of variance (ANOVA) using GraphPad Prism 8.0 to compare the means between the different treatment groups. Each experiment was performed three times and the average values obtained from these replicates were calculated. The results were presented as the mean ± standard error of the mean (SEM). Differences were considered statistically significant at *P* < 0.05.

## 3 Results

### 3.1 Effects of DHA on the motility and kinematics

**Figure 1:**
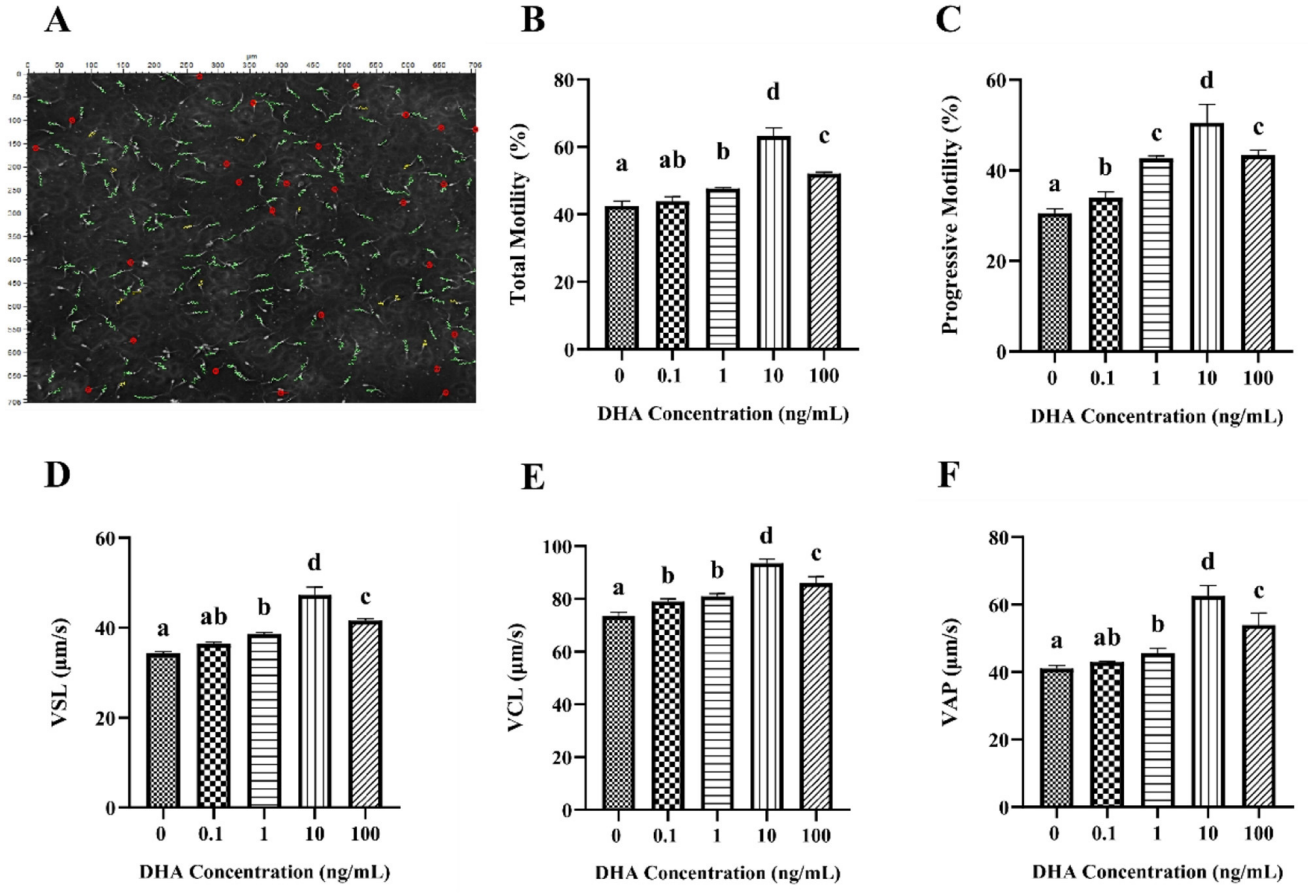
Effects of various concentrations of DHA on the motility and kinematic properties of frozen-thawed yak sperm. **(A)** The motility tracks of yak spermatozoa, as recorded by the CASA system. The green line represents progressive motility, the yellow line represents local motility, and the red dot represents non-motile or immotile sperm. **(B)** The total motility percentage of each treatment group (%). **(C)** The progressive motility of the sperm samples. **(D)** Straight line velocity (VSL). **(E)** Curvilinear velocity (VCL). **(F)** Average path velocity (VAP). Different lowercase letters above the bars or data represent significant differences (*P* < 0.05) among the treatment groups, while the same letters represent no significance (*P* > 0.05).

The motility and kinematic parameters of frozen-thawed yak semen treated with varying concentrations of DHA are shown in Figure 1. The sperm tracks were captured and analyzed using the CASA system (Figure 1A). As shown in Figure 1B, the total motility of frozen-thawed yak sperm across all treatment groups exceeded 40%. Notably, the addition of 1, 10, and 100 ng/ml DHA to the semen extenders significantly enhanced the total motility of yak sperm compared to the control group (*P* < 0.05), with the 10 ng/ml group exhibiting the highest motility, followed by the 100 ng/ml group (*P* < 0.05). However, there was no significant difference in total motility between 0.1 ng/ml DHA treatment and the control group (*P* > 0.05). The progressive motility was significantly increased by the addition of DHA to the semen extender compared to the control group (*P* < 0.05), with the 10 ng/ml DHA group showing the highest progressive motility, followed by the 1 and 100 ng/ml groups (*P* < 0.05, Figure 1C).

The kinematic of frozen-thawed yak spermatozoa treated with DHA is presented in Figures 1D–F. The addition of 1, 10, and 100 ng/ml DHA to the semen extender significantly improved the VSL, VCL, and VAP of the frozen-thawed yak spermatozoa compared to the control group (*P* < 0.05). The 0.1 ng/ml DHA treatment did not significantly affect VSL and VAP (*P* > 0.05) but predominantly improved the VCL than that of the control group (*P* < 0.05).

### 3.2 Effects of DHA on the acrosome integrity

**Figure 2:**
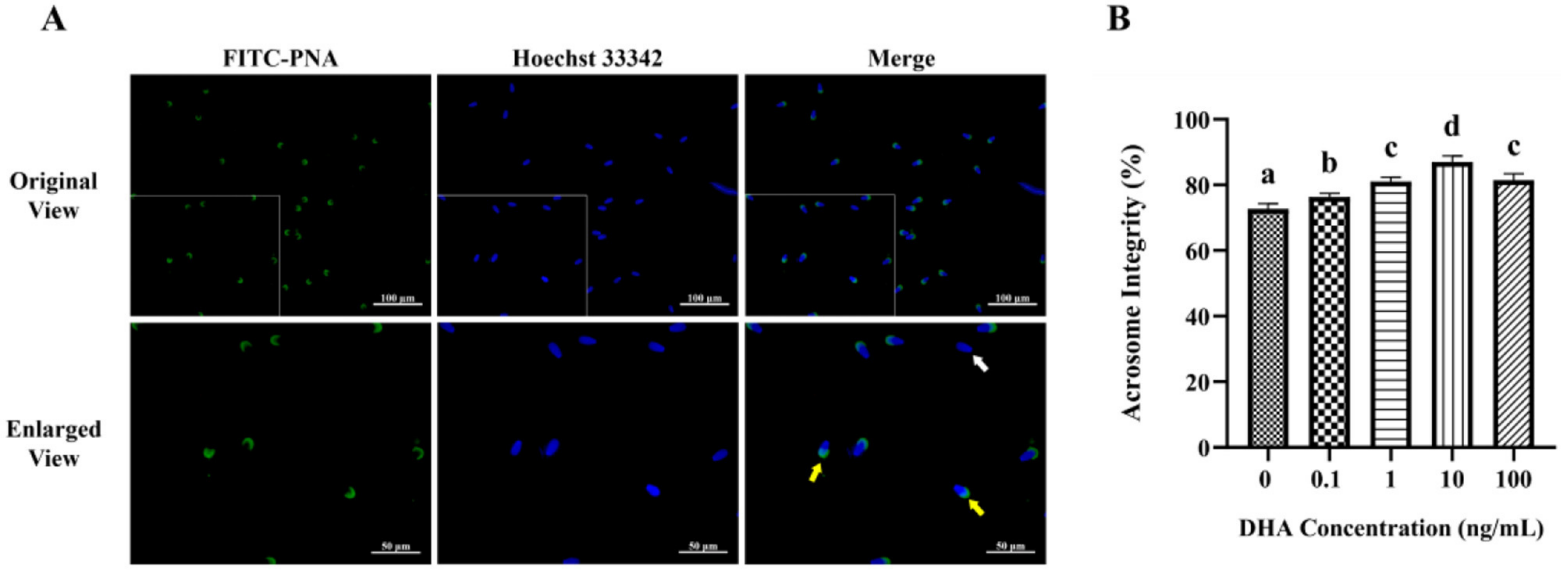
Effects of various concentrations of DHA on the acrosome integrity of frozen-thawed yak sperm. **(A)** The acrosome integrity of yak sperm detected by FITC-PNA fluorescence staining. The yellow arrow represents sperm cells with an intact acrosome, while the white arrow represents sperm cells with a damaged acrosome. **(B)** The ratio of sperm cells with intact acrosome. Different lowercase letters above the bars or data represent significant differences (*P* < 0.05) among the treatment groups, while the same letters represent no significance (*P* > 0.05).

To evaluate the acrosome integrity of frozen-thawed yak sperm, the FITC-PNA fluorescence staining was employed, as depicted in Figure 2A. As shown in Figure 2B, the addition of 0.1, 1, 10, and 100 ng/ml DHA to the semen extender significantly improved the acrosome integrity of frozen-thawed yak sperm compared to the control group (*P* < 0.05). Among these treatment groups, the 10 ng/ml DHA group exhibited the highest percentage of sperm with intact acrosome (*P* < 0.05). Nevertheless, there was no significant difference in acrosome integrity between the 1 and 100 ng/ml DHA groups (*P* > 0.05), although both concentrations still resulted in higher intact acrosome ratios compared to the 0.1 ng/ml group (*P* < 0.05). These findings indicate that DHA can have a protective effect on acrosome integrity with an optimal concentration.

### 3.3 Effects of DHA on membrane integrity

**Figure 3:**
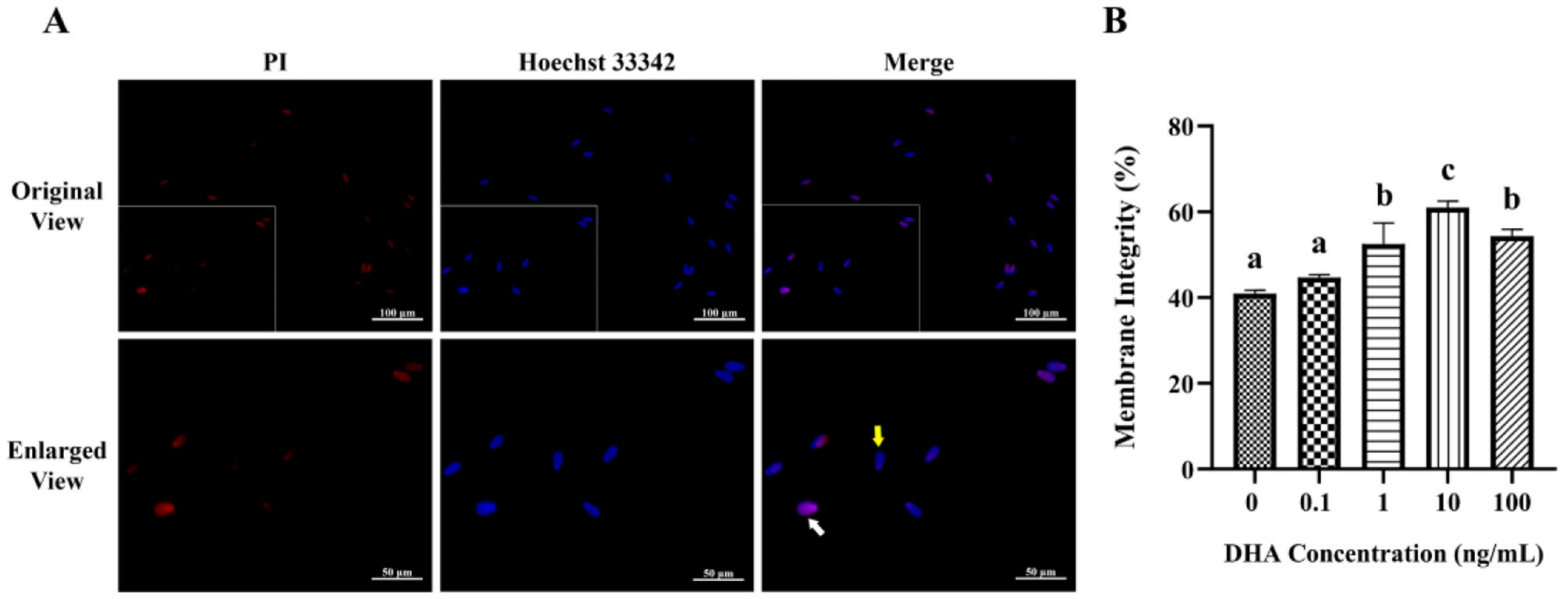
Effects of various concentrations of DHA on the membrane integrity of frozen-thawed yak sperm. **(A)** The membrane integrity as detected by PI/Hoechst 33342 fluorescence staining. The yellow arrow represents the intact membranes, while the white arrow represents the damaged membranes. **(B)** The ratio of spermatozoa with intact membranes. Different lowercase letters above the bars or data represent significant differences (*P* < 0.05) among the treatment groups, while the same letters represent no significance (*P* > 0.05).

The fluorescence staining of the membrane integrity is displayed in Figure 3A. The addition of semen extender with 1, 10, and 100 ng/ml DHA markedly enhanced the frozen-thawed sperm membrane integrity (*P* < 0.05). Among these concentrations, the group treated with 10 ng/ml DHA demonstrated the highest level of plasma membrane integrity (*P* < 0.05, Figure 3B). However, the addition of 0.1 ng/ml DHA to the semen extender did not significantly affect the sperm membrane integrity compared to the control group (*P* > 0.05). This indicates that there may be a threshold concentration below which DHA did not exert a significant protective effect on the sperm membrane.

### 3.4 Effects of DHA on the oxidative status

**Figure 4:**
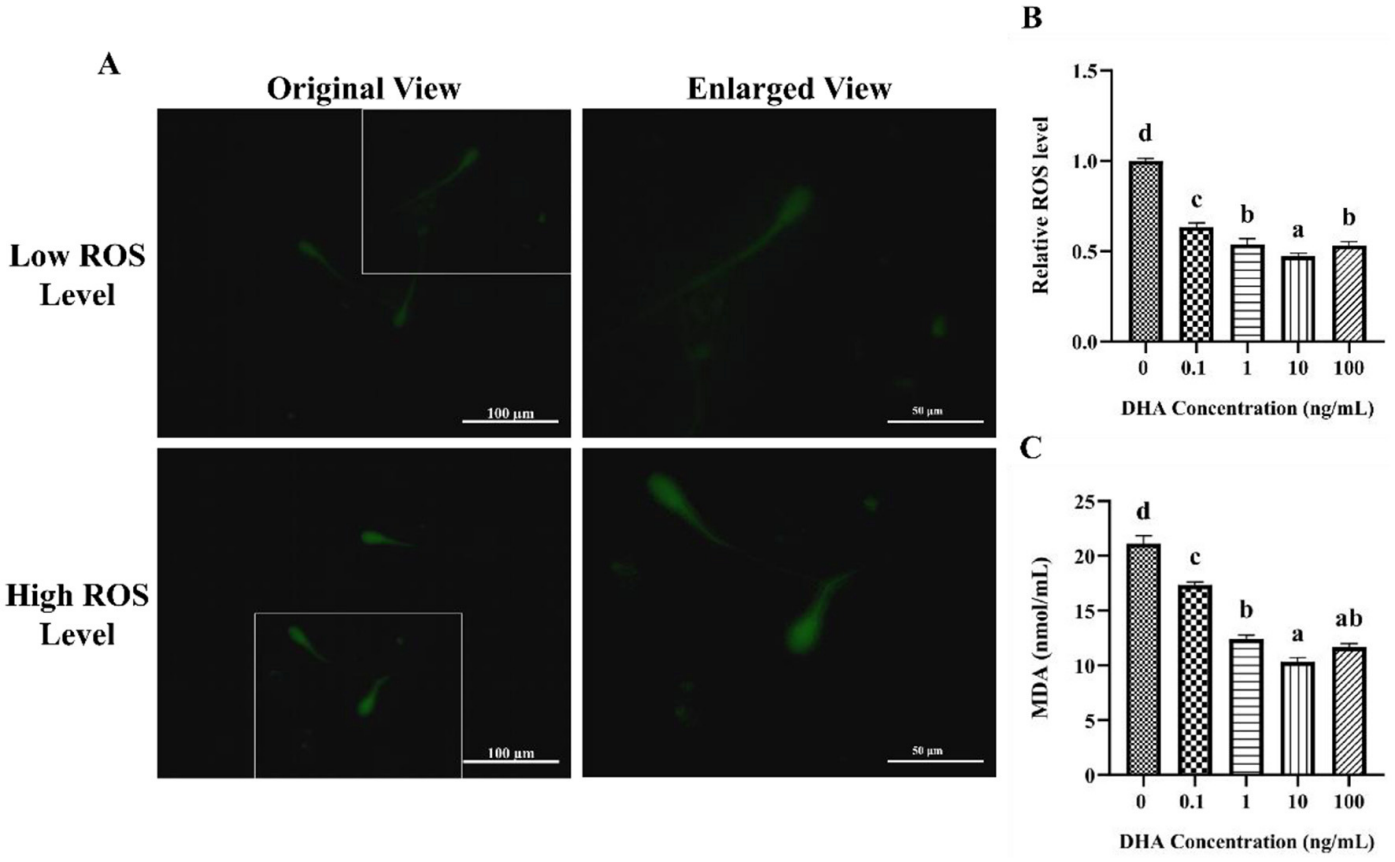
Effects of DHA on the levels of ROS and MDA in yak sperm. **(A)** The ROS staining of yak sperm treatment with various concentrations of DHA. **(B)** The relative ROS levels of all treatment groups. **(C)** The MDA levels in frozen-thawed yak sperm treated with various concentrations of DHA. Different lowercase letters above the bars or data represent significant differences (*P* < 0.05) among the treatment groups, while the same letters represent no significance (*P* > 0.05).

The influence of DHA on the levels of ROS in frozen-thawed yak sperm is depicted in Figure 4. The ROS levels as indicated by the intensity of green fluorescence after treatment with ROS detection reagents are displayed in Figure 4A. The fluorescence intensity corresponded to the ROS levels in frozen-thawed yak sperm, with higher intensity ROS levels. As shown in Figure 4B, the addition of 0.1, 1, 10, and 100 ng/ml DHA to the semen extender significantly reduced the ROS levels in frozen-thawed yak sperm compared to the control group (*P* < 0.05). The 10 ng/ml DHA group exhibited the lowest ROS levels among these treatment groups, indicating that this concentration of DHA was particularly effective at scavenging ROS and mitigating oxidative stress in sperm after cryopreservation (*P* < 0.05). While, there was no significant difference in ROS levels between the 1 and 100 ng/ml treatment groups (*P* > 0.05), although both concentrations still resulted in lower ROS levels compared to the 0.1 ng/ml group (*P* < 0.05).

The MDA content in frozen-thawed yak sperm was shown in Figure 4C, which is a primary product of lipid peroxidation and a marker of oxidative damage. The addition of 0.1, 1, 10, and 100 ng/ml DHA to the semen extender effectively reduced the MDA content (*P* < 0.05), indicating that DHA can protect sperm from lipid peroxidation during cryopreservation. The 10 and 100 ng/ml treatment groups showed even lower MDA levels than the other groups (*P* < 0.05). There was no significant difference in MDA content between the 1 and 100 ng/ml groups (*P* > 0.05), but both concentrations still led to lower MDA levels compared to the 0.1 ng/ml group (*P* < 0.05).

### 3.5 Effects of DHA on the antioxidant capacity

**Figure 5:**
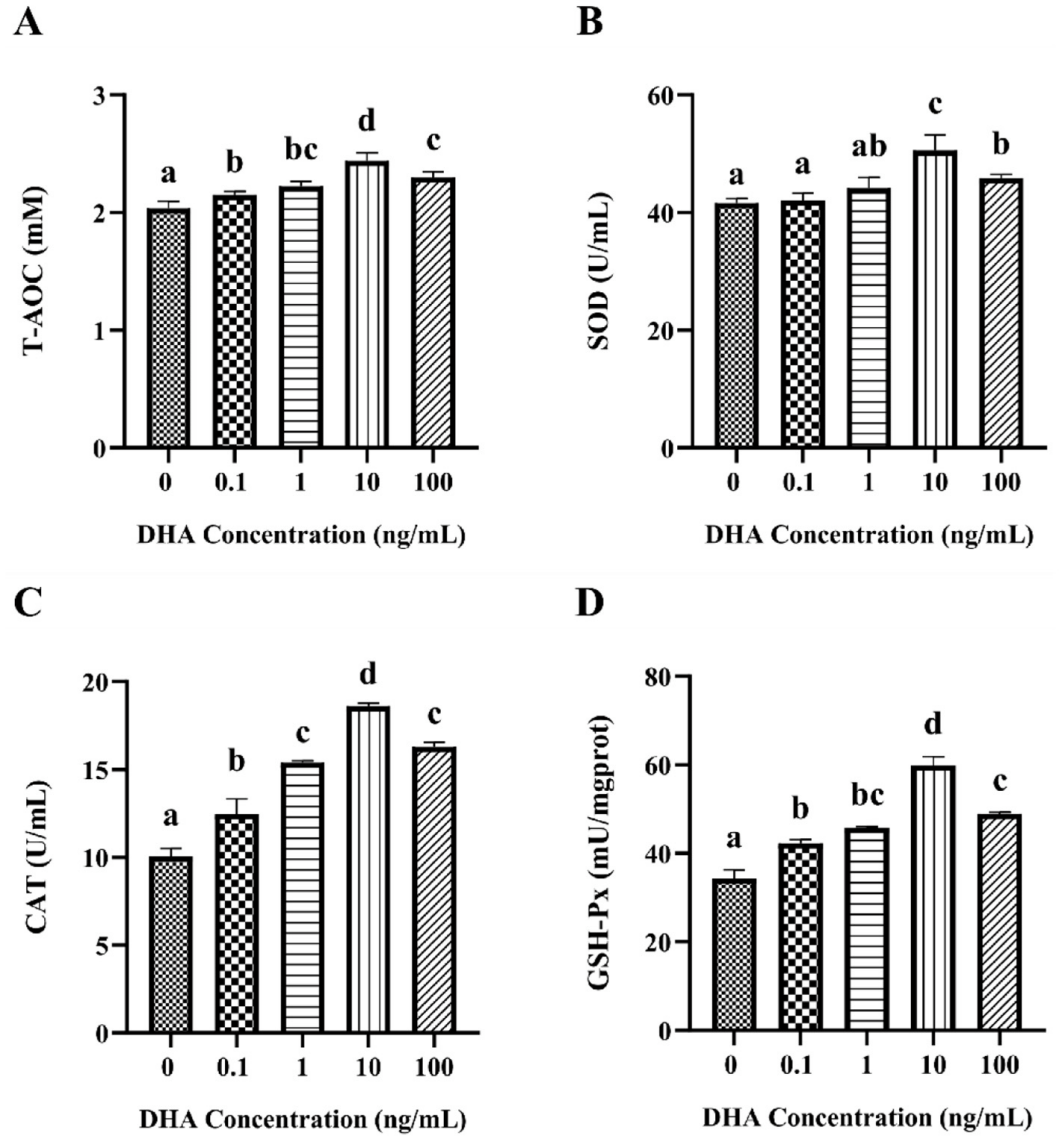
The antioxidant capacity in yak sperm treated with various concentrations of DHA. **(A)** The T-AOC level in the yak sperm. **(B)** The SOD content of the yak sperm. **(C)** The CAT content in the yak sperm. **(D)** The GSH-Px content in the yak sperm. Different lowercase letters above the bars or data points indicate significant differences (*P* < 0.05) among the treatment groups, while the same letters indicate no significance (*P* > 0.05).

To evaluate the antioxidant capacity of frozen-thawed yak sperm following treated with various concentrations of DHA, the T-AOC, SOD, CAT, and GSH-Px contents in yak spermatozoa were assessed (Figure 5). The results indicated that the inclusion of 0.1 ng/ml, 1 ng/ml, 10 ng/ml, and 100 ng/ml DHA in the semen extenders significantly enhanced the T-AOC, CAT, and GSH-Px content of the cryopreserved sperm (*P* < 0.05, Figures 5A, C, D). Regarding the impact on SOD levels, the addition of 0.1 and 1 ng/ml DHA did not significantly affect the relative levels of SOD (*P* > 0.05, Figure 5B). However, increasing the DHA concentration to 10 and 100 ng/ml resulted in significantly higher SOD levels compared to the control group (*P* < 0.05). Most notably, the group treated with 10 ng/ml DHA exhibited the highest levels of all assessed antioxidants compared to other treatment groups (*P* < 0.05).

### 3.6 Effects of DHA on the mitochondrial function

**Figure 6:**
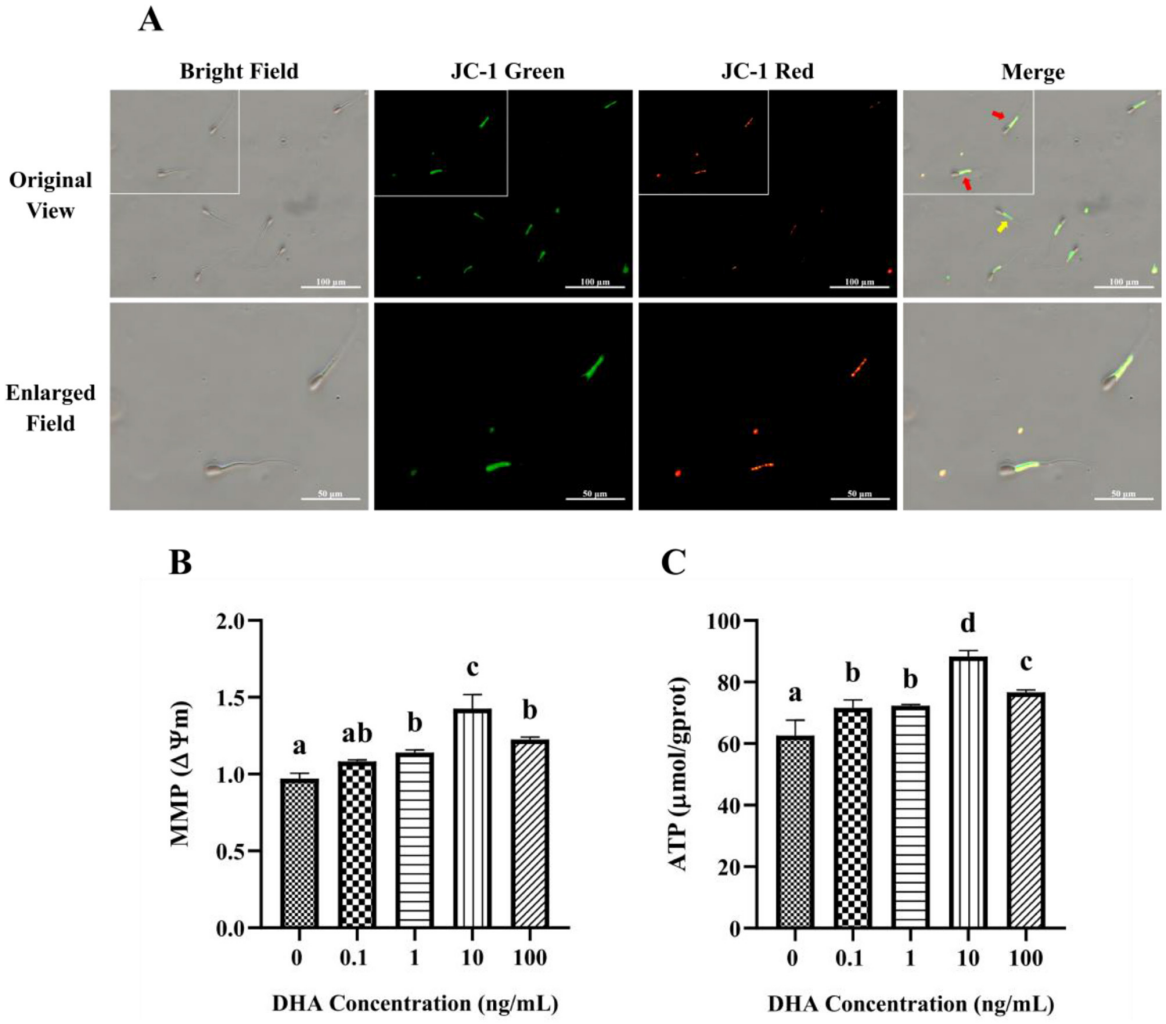
Effects of DHA on the mitochondrial function of yak sperm. **(A)** The JC-1 staining of yak sperm for MMP detection. Red fluorescence represents a high MMP, while green fluorescence represents a low MMP. In the merge field, the red arrow points to sperm with low MMP, and the yellow arrow points to sperm with high MMP. **(B)** The MMP measurement for yak sperm treated with various concentrations of DHA. **(C)** The ATP content in yak sperm from different treatment groups. Different letters above the bars indicate significant differences (*P* < 0.05) among these groups, while the same letters indicate no significant difference (*P* > 0.05).

The MMP in yak semen was assessed using JC-1 fluorescence staining, as depicted in Figure 6A. JC-1 is a sensitive indicator of MMP, with red fluorescence signifying high MMP and green fluorescence indicating low MMP. The addition of 1, 10, and 100 ng/ml DHA to the semen extender had significantly higher MMP levels compared to the control group (*P* < 0.05), and the 10 ng/ml groups exhibited the highest MMP level (Figure 6B). Although there was no significant difference between the 1 ng/ml and 100 ng/ml groups (*P* > 0.05), both showed higher MMP than the control group (*P* < 0.05). In contrast, 0.1 ng/ml DHA did not significantly affect MMP compared to the control group (*P* > 0.05).

The ATP content in sperm from different treatment groups is shown in Figure 6C. Compared with the control group, the addition of DHA to the semen extender significantly improved the ATP content in yak sperm after being frozen-thawed (*P* < 0.05). The 10 ng/ml DHA group had the highest ATP content, suggesting that this concentration is most effective at preserving sperm energy status post-thawed (*P* < 0.05). While there was no significant difference between the 0.1 and 1 ng/ml groups (*P* > 0.05), both concentrations still resulted in higher ATP content compared to the control group (*P* < 0.05).

### 3.7 Effects of DHA on the apoptosis status

**Figure 7:**
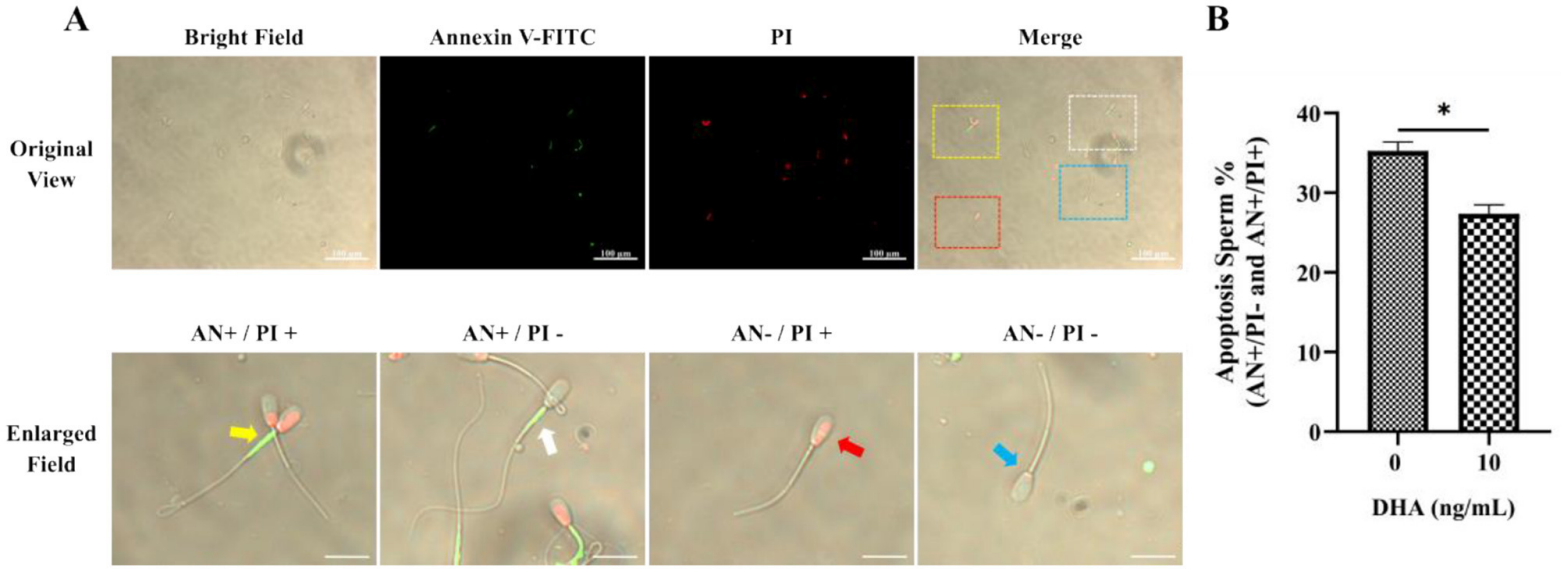
Effects of DHA on the apoptosis status of cryopreserved yak sperm. **(A)** The Annexin V/PI staining was used to determine the apoptosis status of sperm. In the merges field, late apoptotic sperm are indicated by the yellow frame and arrow, early apoptotic sperm by the white frame and arrow, non-viable necrotic sperm by the red frame and arrow, and normal live sperm by the blue frame and arrow. **(B)** The quantitative analysis of apoptotic sperm. The statistical significance is represented by an asterisk (*), indicating *P* < 0.05.

The assessment of apoptosis in frozen-thawed yak semen treated with varying concentrations of DHA was shown in Figure 7A, utilizing Annexin V/PI staining to detect apoptotic spermatozoa. The quantitative analysis of apoptotic spermatozoa, including both early and late stages of apoptosis, in control and 10 ng/ml DHA treatment groups is highlighted in the results (Figure 7B). The data indicates that the inclusion of 10 ng/ml DHA in semen extender markedly reduced the proportion of apoptotic sperm after cryopreservation (*P* < 0.05), suggesting that DHA at this concentration can protect sperm from apoptosis, a form of programmed cell death that can be triggered by the stress of cryopreservation.

### 3.8 Effects of DHA on the protein expression related to apoptosis

**Figure 8:**
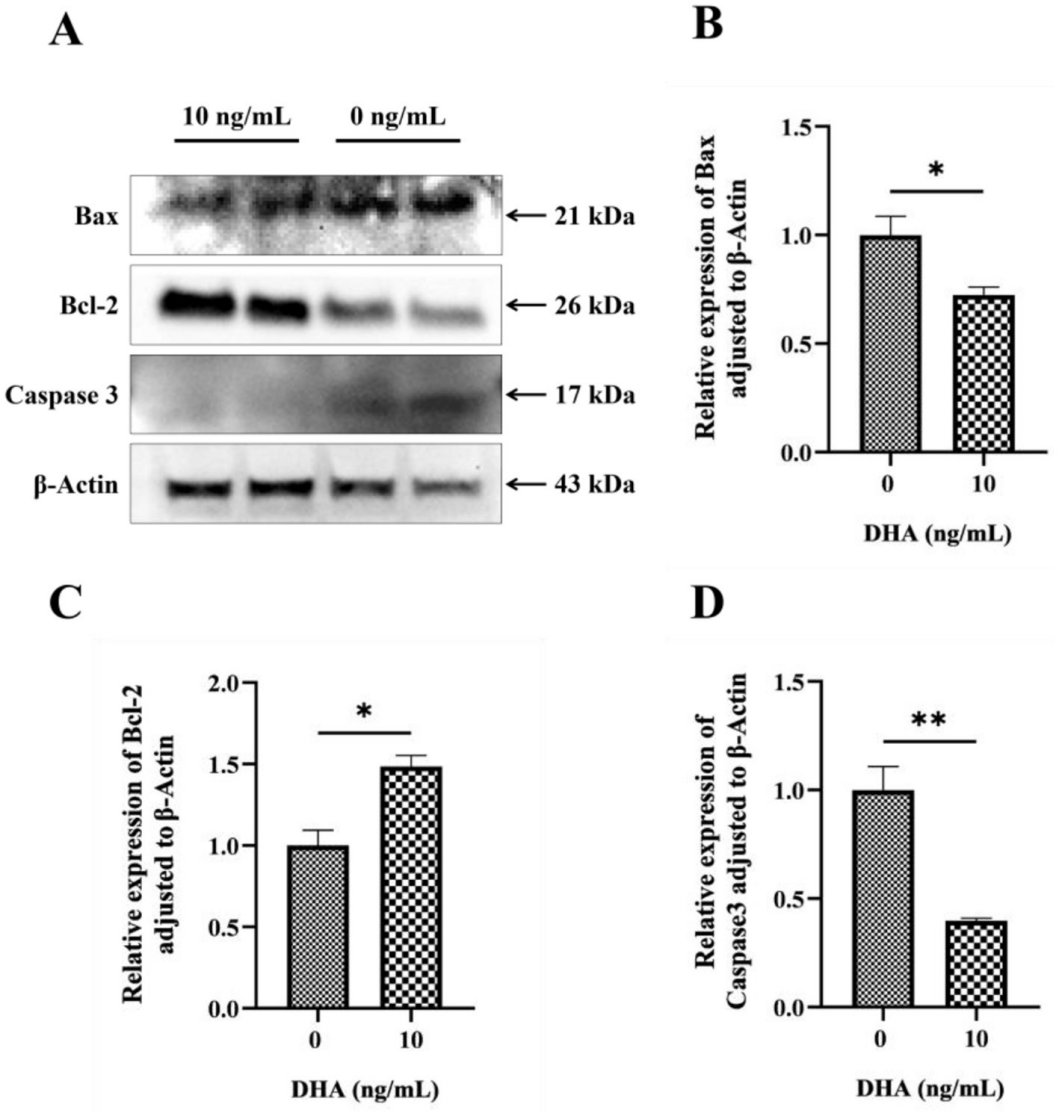
The protein expression levels related to the apoptotic pathway in frozen-thawed yak sperm, were treated with varying concentrations of DHA. **(A)** Western blotting analysis to detect these protein expressions; **(B)** The relative expression level of Bax protein; **(C)** The relative expression level of Bcl-2 protein; **(D)** The relative expression level of Caspase 3 protein. The statistical significance of these differences was indicated by *P* < 0.05, with an asterisk (*) denoting *P* < 0.05 and a double asterisk (**) representing *P* < 0.01.

The effects of DHA on the protein expression levels related to the apoptotic pathway in frozen-thawed yak sperm are shown in Figure 8A. The results showed that the addition of 10 ng/ml DHA to the sperm extender significantly increased the expression level of the anti-apoptotic protein BCl-2 (Figure 8C) after the freezing-thawing process (*P* < 0.05). This suggests that DHA can promote the expression of proteins that inhibit apoptosis, thereby potentially enhancing sperm survival. Conversely, the same concentration of DHA also significantly decreased the expression levels of pro-apoptotic proteins Bax and Caspase3 (*P* < 0.05, Figures 8B, D), indicating that DHA may profit from suppressing the apoptotic pathway in sperm cells during cryopreservation.

## 4 Discussion

As the cornerstone of artificial insemination, semen cryopreservation plays a pivotal role in the conservation and utilization of animal genetic resources. However, the quality and fertilization potential of semen can be significantly compromised following cryopreservation (23). Semen motility and kinematics are key indicators for assessing sperm quality. Previous studies have shown that sperm motility is positively correlated with fertilization rates (24), and only sperm with progressive motility possess the ability to fertilized during natural or artificial insemination (25). In this study, the motility and kinematics of cryopreserved yak semen were significantly improved when treated with the 10 ng/ml DHA in the extender, a finding consistent with previous research on bovines (26). This improvement may be attributed to the increased proportion of PUFAs in sperm dilution due to DHA supplementation, which alleviates the freezing damage and maintains the stability and fluidity of the sperm plasma membrane. This, in turn, protects sperm morphology and internal metabolic homeostasis, thereby preserving sperm motility and kinematics. Nevertheless, research on brown trout has reported an optimal concentration of 7.5 ng/ml, suggesting species-specificity differences in DHA tolerance (27). The sperm plasma membrane contains various long-chain unsaturated fatty acids, including docosahexaenoic acid (DHA), eicosapentaenoic acid (EPA), and arachidonic acid (AA), which are essential for maintaining sperm quality and fertilization capacity (28). Studies on δ6-desaturase (FADS2) knockout mice have indicated that the absence of initial steps in PUFA synthesis can lead to spermatogenesis stasis and infertility (29). However, fertility can be restored in these knockout mice with DHA supplementation (30), confirming the essential role of DHA in sperm structure formation and stability. PUFAs are primarily concentrated in the sperm head and change with capacitation to support fertility (31). The acrosome, a vesicular structure at the sperm head's apex, is integral to the acrosome reaction and fertilization. Acrosin is released from the acrosome to dissolve the oocyte's corona radiate and zona pellucida upon sperm-oocyte binding, ensuring fertilization (32). This study found that the DHA supplementation in the yak sperm extender effectively increased the integrity of the plasma membrane and acrosome after freezing-thawing, with the highest performance in the 10 ng/ml treatment group. This may be attributed to the protective effect of DHA on membrane PUFAs, maintaining a consistent PUFAs ratio and membrane skeleton stability. The results were consistent with those from goat studies but different from those in boar (5 nM) (33, 34), possibly due to the species differences in PUFAs types and compositions on sperm plasma membrane, influencing DHA adaptation and sperm freezing tolerance. Notably, at a high DHA dosage (100 ng/ml), sperm motility, kinematic parameters, and structure integrity were all reduced, indicating a dose-dependent effect of DHA during the yak sperm cryopreservation. High concentration of DHA may have a negative effect, potentially due to alterations in sperm membrane osmosis, fluidity, and permeability.

ROS are single-electron oxygen reduction products in the mitochondrial ETC, resulting from substantial electron leakage at specific oxygen reduction centers (35). During cryopreservation, ROS accumulate, inducing oxidative stress and damaging the sperm's antioxidant system. The sperm plasma membrane, rich in PUFAs, is susceptible to ROS attack, leading to lipid peroxidation (LPO) and formation of the toxic lipid peroxidation products (36, 37). Studies have shown that DHA can significantly reduce ROS levels in rat astrocytes and mice liver (38). This study demonstrated that DHA supplementation in the semen extender effectively reduced the ROS and MDA content in frozen-thawed yak sperm, particularly in the 10 ng/ml DHA group, where ROS levels and MDA content were significantly lower than in other treatment groups. These positive effects may be due to DHA's efficient scavenging of ROS, alleviating damage to antioxidant enzyme structure caused by MDA. Other research has indicated that the combination of DHA and mitochondrial membrane can maintain membrane composition and structure, affecting the opening of mitochondrial permeability transition pore (mPTP) and reducing the proton leakage in the ETC, ultimately decreasing ROS content (39). Notably, 100 ng/ml DHA increased ROS and MDA compared to the 10 ng/ml treatment group, possibly because high DHA concentrations act as pro-oxidants, facilitating ROS generation. Previous studies have also shown that DHA can increase ROS and lipid peroxide concentration, triggering cancer cell apoptosis (40). Therefore, a balance between DHA concentration and sperm antioxidant status should be considered when using DHA as an antioxidant. During spermatogenesis, endogenous antioxidants, including SOD, CAT, and GSH-Px, are present in both seminal plasma and spermatozoa (41, 42), forming the total antioxidant system and reflecting sperm antioxidant capacity (43, 44). SOD directly catalyzes the conversion of superoxide anions to oxygen and water, playing a key role in reducing oxidative stress damage; CAT catalyzes hydrogen peroxide anions to water and oxygen, while GSH-Px reduces peroxide to hydroxyl compounds, decreasing peroxide damage to plasma membrane structure and function (45). Normally, the endogenous antioxidant system protects sperm from oxidative stress, but the rapid temperature drop during cryopreservation leads to the inability of endogenous antioxidants to meet sperm antioxidant requirements, necessitating exogenous antioxidants. This study found that DHA supplementation in the sperm extender significantly increased the SOD, CAT, and GSH-Px levels, with each antioxidant reaching its maximum at a DHA dosage of 10 ng/ml. These results were similar to those from bull studies, where 10 ng/ml DHA supplementation significantly increased frozen-thawed bull sperm SOD levels (46). Another study showed that DHA could reduce ROS and MDA levels in PC12 cells and improve antioxidant activity (47), supporting the current findings. However, at a dosage of 100 ng/ml, SOD, CAT, and GSH-Px activities were significantly decreased, possibly due to high DHA concentration altering yak sperm osmosis and internal homeostasis, affecting antioxidant activity.

Mitochondria, as energy centers, maintain sperm metabolism and motility by generating ATP (48, 49). ATP affects linear sperm movement by influencing tail movement, which in turn affects progressive sperm movement from the uterine to the oviduct, essential for sperm motility and fertilization (50). MMP is a key indicator of mitochondrial activity, with only mitochondria with high MMP processing biological activity. However, ROS can be generated by electron-oxygen reaction at complexes I and III of the mitochondrial ETC and accumulate in mitochondria (51), altering membrane permeability and decreasing MMP (5). The mPTP, a transmembrane protein on the mitochondrial inner membrane, opens in response to oxidative stress (52), initiating permeability swelling, MMP decrease, and the release of Ca^2+^ and proapoptotic factors from mitochondria, leading to apoptosis and necrosis (53). Studies on rats have shown that DHA can increase SOD and CAT activities, improving the mitochondrial stability and tolerance to oxidative stress (54). Another study revealed that high ROS levels can induce lower MMP in ejaculated sperm, leading to infertility (55). This study found that DHA supplementation in semen extender significantly increased ATP levels and MMP in frozen-thawed yak sperm, with the best results at a 10 ng/ml DHA treatment. This may be due to DHA regulating the mitochondrial respiratory chain in yak sperm, improving mitochondrial function, and decreasing ROS generation, thereby reducing ROS attacks on the mitochondrial membrane.

Mitochondrial damage triggered by excessive ROS can activate the apoptosis pathway. At high ROS levels, the MAPK signaling pathway is activated, and Bax and Bak mediate the formation of mPTP at the mitochondrial outer membrane and depolarization of the inner membrane, causing the release of proapoptotic factors such as CytC from mitochondria into the cytoplasm. Then CytC combines with the Apaf1, activating the caspase signaling pathway and initiating apoptosis (56). The mitochondrial apoptotic pathway is regulated by the Bax/Bcl-2 ratio. As an anti-apoptotic protein, Bcl-2 can inhibit CytC release into the cytoplasm to prevent apoptosis occurrence; while Bax could bind to Bcl-2 to prevent apoptosis, while Bax can bind to Bcl-2 to neutralize its anti-apoptotic function. Therefore, the Bax/Bcl-2 ratio is often used to measure apoptosis status (57). In this study, 10 ng/ml DHA supplementation in semen extender significantly reduced the proportion of apoptotic yak sperm after freezing-thawing compared to the control group. Further analysis of apoptosis-related protein expression revealed that 10 ng/ml DHA significantly increased anti-apoptotic protein Bcl-2 expression and inhibited proapoptotic protein Bax and Caspase3 levels. This may be due to DHA reducing ROS generation and modulating mitochondrial function to alleviate mitochondrial membrane structure disruption caused by ROS. Moreover, DHA effectively maintained mitochondrial membrane structure stability, preventing the release of proapoptotic factors from sperm mitochondria into the cytoplasm, ultimately blocking mitochondria-mediated apoptosis. However, the specific signaling pathways through which DHA regulates mitochondrial function require further investigation.

## 5 Conclusion

**Figure 9:**
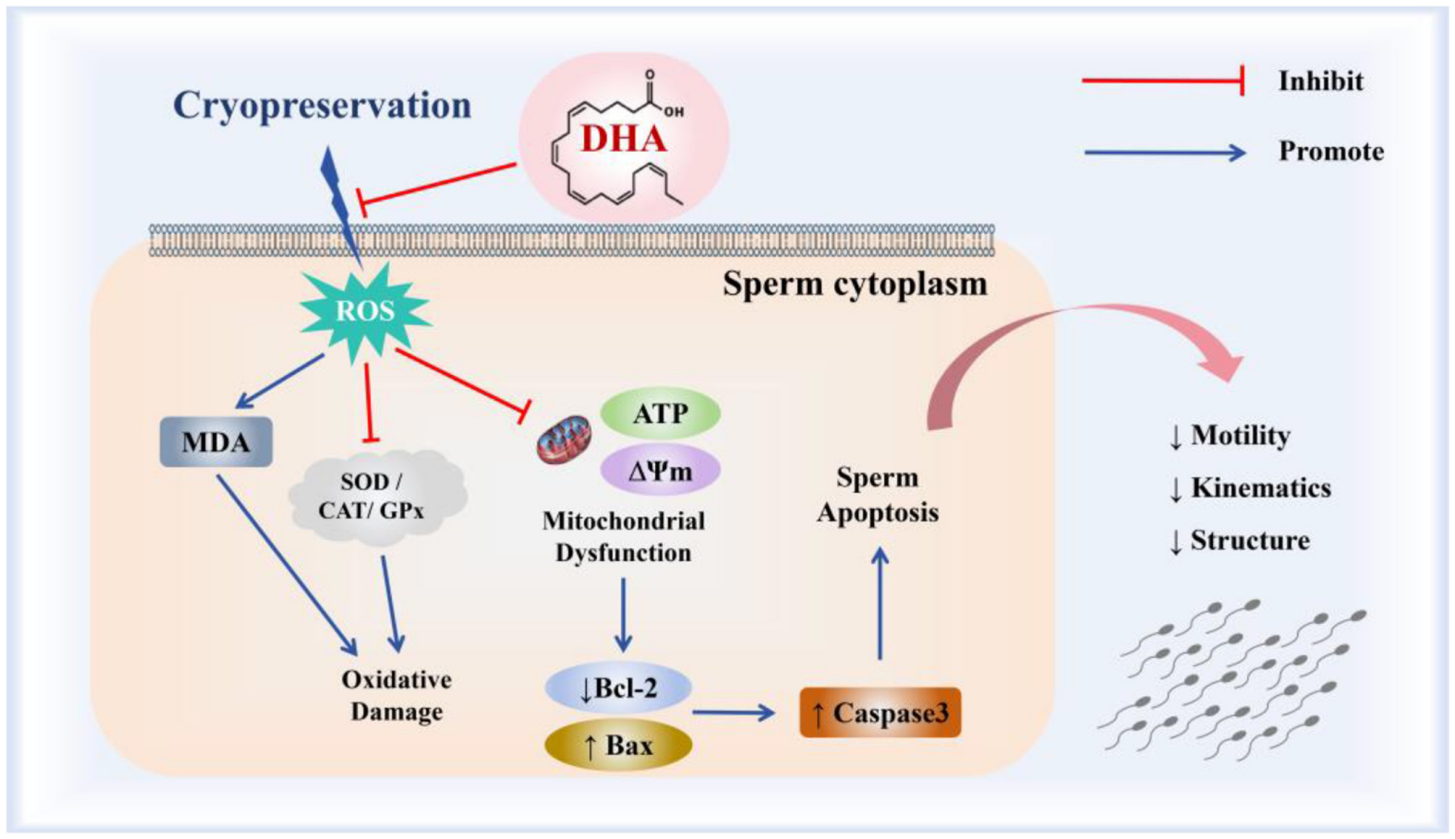
The mechanism of DHA on the quality of yak spermatozoa after cryopreservation.

Taken together, the incorporation of 10 ng/ml DHA into the semen extender notably enhanced the quality of cryopreserved yak spermatozoa via mitigating oxidative damage, bolstering the activities of antioxidant enzymes, and augmenting mitochondrial functions, thereby preventing the apoptotic pathway in yak spermatozoa (Figure 9). Future research will focus on evaluating the fertilization potential of the cryopreserved yak semen treated with DHA and exploring the observed valuable insights into refining cryopreservation techniques for yak semen and optimizing the use of DHA as a cryoprotectant across different species.

## Data Availability

The raw data supporting the conclusions of this article will be made available by the authors, without undue reservation.
